# Retraction: Mutual Regulation of Bcl-2 Proteins Independent of the BH3 Domain as Shown by the BH3-Lacking Protein Bcl-x_AK_

**DOI:** 10.1371/journal.pone.0314638

**Published:** 2024-11-22

**Authors:** The PLOS ONE Editors

After this article [1] was published, concerns were raised about results presented in Figs 3, 4, 6, and 7. Specifically:

- The following panels appear similar:
  ○ The Fig 3A Bak, Bax and ꞵ-actin panels of this article [1] and the Fig 3A Bak, Bax and ꞵ-actin panels of [2].
  ○ The Fig 6A Mel-2a VDAC panel and the Fig 6A A357-Bcl-2 VDAC panel.
  ○ The Fig 6A A375 Mock Bcl-x_AK_ panel and the Fig 6A A375 Bcl-2 Bcl-x_AK_ panel.
  ○ The Fig 6B Mel-2a Bak panel and the Fig 6B A357-Bcl-2 VDAC panel when flipped vertically.
  ○ The Fig 7A Bcl-2 panel of this article [1] and the Fig 5A Bcl-2 panel of [2] despite being used to represent results obtained from different cell lines.
- The Fig 4A Control DU145 Bax-EGFP and Bcl-x_AK_ OFF DU145 Bax-EGFP panels appear to partially overlap.

The corresponding author responded to queries about the above, stating that they were unable to provide the raw data. In the absence of the raw data the issues with these figures cannot be resolved.

In light of the above concerns which question the reliability of the published results, the *PLOS ONE* Editors retract this article.

JE agreed with the retraction. MP, AMH, BG, PTD, and ES either did not respond directly or could not be reached.
